# Adaptive Diagnosis for Rotating Machineries Using Information Geometrical Kernel-ELM Based on VMD-SVD

**DOI:** 10.3390/e20010073

**Published:** 2018-01-21

**Authors:** Zhipeng Wang, Limin Jia, Yong Qin

**Affiliations:** 1 State Key Lab of Rail Traffic Control and Safety, Beijing Jiaotong University, Beijing 100044, China; 2 National Engineering Laboratory for System Safety and Operation Assurance of Urban Rail Transit, Guangzhou 510000, China; 3 Beijing Research Center of Urban Traffic Information Sensing and Service Technologies, Beijing Jiaotong University, Beijing 100044, China

**Keywords:** fault diagnosis, information geometry, kernel extreme learning machine, variation mode decomposition

## Abstract

Rotating machineries often work under severe and variable operation conditions, which brings challenges to fault diagnosis. To deal with this challenge, this paper discusses the concept of adaptive diagnosis, which means to diagnose faults under variable operation conditions with self-adaptively and little prior knowledge or human intervention. To this end, a novel algorithm is proposed, information geometrical extreme learning machine with kernel (IG-KELM). From the perspective of information geometry, the structure and Riemannian metric of Kernel-ELM is specified. Based on the geometrical structure, an IG-based conformal transformation is created to improve the generalization ability and self-adaptability of KELM. The proposed IG-KELM, in conjunction with variation mode decomposition (VMD) and singular value decomposition (SVD) is utilized for adaptive diagnosis: (1) VMD, as a new self-adaptive signal processing algorithm is used to decompose the raw signals into several intrinsic mode functions (IMFs). (2) SVD is used to extract the intrinsic characteristics from the matrix constructed with IMFs. (3) IG-KELM is used to diagnose faults under variable conditions self-adaptively with no requirement of prior knowledge or human intervention. Finally, the proposed method was applied on fault diagnosis of a bearing and hydraulic pump. The results show that the proposed method outperforms the conventional method by up to 7.25% and 7.78% respectively, in percentages of accuracy.

## 1. Introduction

As industrial systems become more and more sophisticated, slight faults may result in catastrophes. Therefore, fault diagnosis technology has become more and more significant for the safety and reliable utilization of systems [1]. As we known, failures of rotating machineries are common causes of breakdown in industries. The growing demand for safety and reliability in industries requires a smart fault diagnosis system for rotating machineries [2]. Many researchers have studied the implementations of fault diagnosis algorithms on mobile devices for wider adoption [3,4,5,6]. Not only can the efficient fault diagnosis keep the monitored systems healthy and safe, they can also decrease the cost of repairs or replacements [7].

A lot of research have been done on the fault diagnosis for rotating machineries during the past decades. Vibration analysis is the main method for condition monitoring of rotating machineries [8]. Currently, a lot of data-driven methods have been proposed, for example, fuzzy logic [2,8], principal component analysis [9,10]. Most of the methods mainly require certain and stable operation conditions. However, it is known that real-world machineries are usually operating under variable and uncertain conditions. Sometimes, the undergoing operation condition is even unknown. It is quite challenging that a diagnosis model trained under condition A is used under another unknown condition B. Misdiagnosis will emerge considerably in this situation.

Many studies have been done on this subject. Yu et al. [10] proposed a hybrid feature selection method to deal with it, but required prior knowledge about the undergoing conditions. Liu et al. [11] and Tian et al. [12] employed multifractal detrended fluctuation analysis and permutation entropy to diagnose bearing faults under fluctuations in operation conditions, but required human intervention for the final clustering. Tian et al. [13] employed extreme learning machine for bearing fault diagnosis under variable conditions, but required training data under all conditions. However, it is even impossible to acquire such prior knowledge and training data under all possible operation conditions. That is because the real undergoing operation conditions are often unknown and uncertain. Therefore, it is valuable to develop a new fault diagnosis method which can adapt itself to unknown variable conditions with little prior knowledge or human intervention. In this paper, it is described as “adaptive diagnosis”.

An adaptive diagnosis method should be provided with two characteristics:

(1) Adaptivity to unknown variable conditions. Rotating machineries often work under unknown variable conditions without any prior knowledge or training data. Since the health characteristics of rotating machineries might be variable under different conditions, an adaptive diagnosis method ought to be self-adaptive to unknown variable conditions.
(2) Automaticity with little human intervention. An adaptive diagnosis method should be applied automatically and independent of human intervention as much as possible.

Generally, if a fault diagnosis method satisfies the above characteristics, it can be considered as an adaptive diagnosis method. The application of adaptive diagnosis methods can not only reduce the dependence on operators’ experiences and skills, but also decrease the complexity and cost of condition monitoring systems. This paper attempts to propose an adaptive diagnosis method for rotating machineries by using vibration analysis.

Feature extraction is the basic step during fault diagnosis. It should be self-adaptive and provides remarkable features with little human intervention to extract valid features under variable conditions. Currently, a lot time-frequency analysis methods have been employed for feature extraction for rotating machineries, such as bearing, gearbox [14,15,16,17,18,19,20,21,22]. Wavelet transform, as a well-known time-frequency analysis tool, has been employed wildly to decompose the nonstationary signals. However, it still suffers several unacceptable disadvantages [23,24,25]. It is considered as an improved Fourier transform with adjustable windows [26]. The structure of the wavelet basic function is permanent during the decomposition [23,27]. Obviously, its non-adaptive nature is not appropriate for adaptive diagnosis.

Empirical mode decomposition (EMD), proposed by Huang et al. [28] is another well-known method and can decompose signals to several intrinsic mode functions (IMFs) according to the time and scale characteristics. It is completely self-adaptive and seems suitable for adaptive diagnosis [28,29,30,31,32]. However, is suffers from many problems, such as: modal aliasing, pseudo components, end effect and so on. Therefore, a more efficient self-adaptive method is needed.

Variation mode decomposition (VMD), as a novel self-adaptive method, was proposed by Dragomiretskiy et al. in 2014 [33]. It is a non-recursive method to decompose signals into quasi-orthogonal IMFs, each with a center frequency. It has been proved to overcome the problems of EMD and its extended methods [34]. Therefore, VMD is employed for feature extraction.

The IMFs acquired by VMD can be used to form a matrix, which is too large to be directly used for fault diagnosis. Therefore, a suitable algorithm is needed to extract the intrinsic characteristics of the matrix. Singular value decomposition (SVD), which has been proven to extract the features to periodic impulses is employed to obtain intrinsic features from the matrix with favorable stability [35,36].

After feature extraction, the next step is fault clustering. As aforementioned, the fault clustering method should adapt to unknown variable conditions automatically with little prior knowledge or human intervention. Therefore, the traditional discriminant analysis methods, such as Mahalanobis distance [37,38,39] and Fisher discriminant analysis [40,41], are not suitable, because they mainly require certain levels of expertise and threshold settings. In contrast, computation intelligence techniques are preferred in this situation [42]. The computation intelligence techniques are data-driven and have been employed broadly in fault diagnosis, such as: Bayes net classifier [43], optimization algorithms [44,45] and artificial neural networks [46,47,48]. Deep learnings, such as efficient and remarkable algorithms have been employed automatically with little human intervention [49,50,51]. They are capable to even extract feature self-adaptively and seems suitable for adaptive diagnosis. Deep learnings are data-hungry and require plenty of training data which are hardly acquired in practice, especially the faulty data under different conditions.

Extreme learning machine (ELM), proposed by Huang [52,53], has been proven as an efficient algorithm for regression and multi-classification [54]. Compared with the conventional gradient-based algorithms and support vector machine (SVM), ELM spends less running time and performs better [55]. On this basis, Kernel-ELM (KELM) [56] is proposed by using a kernel function to improve the generalization ability and reduce possible over-fitting problems.

Similar to SVM, the performance of KELM also relies on the kernel function. But the choosing of the kernel function mainly depends on prior knowledge and expertise [55,56,57]. However, different kernel types or parameters maybe only fit several specific datasets respectively. Whereas measured signals under different conditions may prefer different kernel types or parameters. Therefore, it is necessary to modify the algorithm self-adaptively, to make sure that the diagnosis method is insensitive enough to the manual configuration of kernel and performs acceptably even if the kernel types or parameters are set badly.

Information geometry (IG), proposed by Amari [58], which aims to analyze information theory, statistics and machine learning based on differential geometry, offers a feasible approach. It can analyze and modify machine learning algorithms by convex analysis and constructing differential manifolds. By elucidating the dualistic differential-geometrical structure, information geometry has been widely applied [59,60,61,62,63,64]. 

In this paper, motivated by information geometry, we propose a novel algorithm, information geometrical kernel-ELM (IG-KELM). Firstly, we specify the geometrical structure and Riemannian metric of Kernel-ELM from the perspective of information geometry. Then, a data-dependent conformal transformation is created with Mahalanobis distance to modify the KELM self-adaptively. IG-KELM is insensitive to inappropriate kernel configuration, and can adapt itself to signals under variable conditions with little prior knowledge or human intervention. The feasibility and effectivity of IG-KELM was verified by simulation experiments.

The outline of this paper is as follows: Section 2 introduces VMD, Kernel-ELM, Riemannian metric of Kernel-ELM, information geometrical kernel-ELM, as well as the scheme of the proposed method; Section 3 describes the simulation experiment performed to verify IG-KELM; Section 4 describes the applications of the proposed method on fault diagnosis for bearing and hydraulic pump; and Section 5 is the conclusions.

## 2. Methodology

As aforementioned, VMD is the core algorithm for feature extraction, and kernel-ELM is the basic algorithm for fault clustering. In this section, VMD and kernel-ELM is introduced firstly. Then, the analysis of Riemannian metric of Kernel-ELM is described. On this basis, the IG-KELM is proposed. At the end of this section, the scheme of adaptive diagnosis based on VMD-SVD and IG-KELM is described.

### 2.1. Variational Mode Decomposition

VMD is capable to estimate the modes and determine the correlative bands of fault feature at the same time, which can decompose the signal into several IMFs [33]. VMD is a constrained variational problem represented by the following equation:

(1) $$\begin{array}{c} \underset{\mu_{k},\omega_{k}}{\min}\left\{{\sum_{k}\left\|\partial_{t}[(\sigma_{t}+\frac{j}{\pi t})\mu_{k}(t)]e^{-jt\omega_{k}}\right\|}_{2}^{2}\right\},\\ \mathrm{subject}\;\mathrm{to}\;\sum_{k}\mu_{k}=f \end{array}$$

where $\mu_{k}$ and $\omega_{k}$ are IMF components and their center frequencies.

To obtain the optimal solution of the problem, introduce the augmented Lagrange function:

(2) $$L\left(\mu_{k},\omega_{k},\lambda \right)=\alpha {\sum_{k}\left\|\partial_{t}[(\sigma_{t}+\frac{j}{\pi t})\mu_{k}(t)]e^{-jt\omega_{k}}\right\|}_{2}^{2}+{\left\|f-\sum \mu_{k}\right\|}_{2}^{2}+\langle \lambda ,f-\sum \mu_{k}\rangle .$$

The mode number k and quadratic penalty α are set in advance, while the sub-mode function $\mu_{k}{}^{1}$, the center frequency $\omega_{k}{}^{1}$ and the Largrangian multiplier $\lambda^{1}$ are initialized [33]. Then modes $\mu_{k}{}^{}$ and the center frequency $\omega_{k}$ are renewed respectively by Equations (3) and (4):

(3) $$\mu_{k}{}^{n+1}\leftarrow \frac{\overset{\wedge }{f}-\sum_{i<k}{\hat{\mu }}_{i}{}^{n+1}-\sum_{i>k}{\hat{\mu }}_{i}{}^{n}+\frac{{\hat{\lambda }}^{n}}{2}}{1+2\alpha {\left(\omega -\omega_{k}{}^{n}\right)}^{2}},k\in \left\{1,K\right\},$$

(4) $$\omega_{k}{}^{n+1}\leftarrow \frac{\int_{0}^{\infty }\omega {\left|{\overset{⌢}{\mu }}_{k}{}^{n+1}\left(\omega \right)\right|}^{2}d\omega }{\int_{0}^{\infty }{\left|{\overset{⌢}{\mu }}_{k}{}^{n+1}\left(\omega \right)\right|}^{2}d\omega },k\in \left\{1,K\right\}.$$

After the modes and center frequencies are updated, the Largrangian multiplier *λ* is also updated by Equation (5):

(5) $$\lambda^{n+1}=\lambda^{n}+\tau \left(x-\sum_{k}\mu_{k}{}^{n+1}\right),$$

$\mu_{k}$, $\omega_{k}$ and *λ* are updated iteratively until Equation (6) is satisfied.

(6) $$\sum_{k}{\left\|\mu_{k}{}^{n+1}-\mu_{k}{}^{n}\right\|}_{2}^{2}/{\left\|\mu_{k}{}^{n}\right\|}_{2}^{2}<\varepsilon .$$

### 2.2. Kernel-ELM

**Figure 1:**
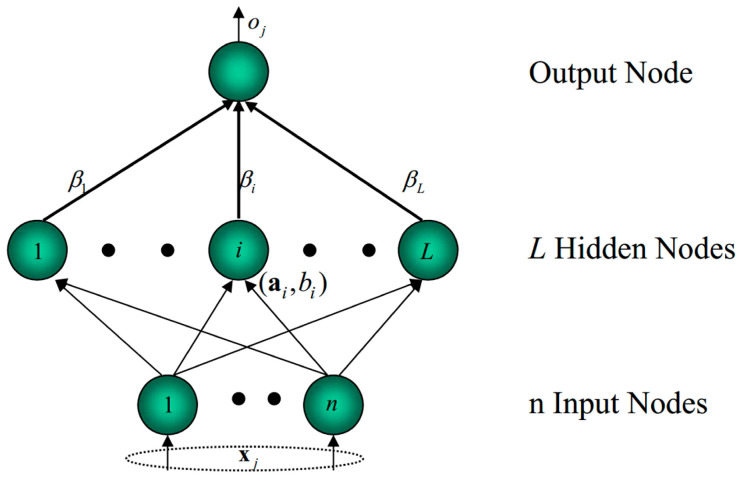
The architecture of ELM.

ELM is originally developed for the training of Single hidden layer feedforward networks (SLFNs) and then extended to the generalized SLFNs. The architecture of ELM is shown in Figure 1. The details about ELM can be found in Huang et al. [52,53]. Consider a dataset $\left(x_{i},y_{i}\right)$, where $x_{i}\left(i=1,2,\cdots ,N\right)$ is input vector, $y_{i}\left(i=1,2,\cdots ,M\right)$ is output vector. The output function of ELM can be described as:

(7) $$f_{L}(x)=\sum_{i=1}^{L}\beta_{i}G\left(\alpha_{i},b_{i},x\right)=h(x)\cdot \beta ,$$

where, *L* is the number of hidden neurons, $\alpha_{i}$ is the weight vector connecting the *i*th hidden node and the input nodes, $b_{i}$ is the bias of the *i*th hidden node, $\beta_{i}$ is the weight vector connecting the *i*th hidden node and the output nodes, G(•) represents the hidden nodes activation function. h(x) is the hidden layer output matrix of the network.

According to the ELM, $\alpha_{i}$ and $b_{i}$ are randomly assigned and the least square solution of *β* is computed by the following objective function:

(8) $$\{\begin{array}{c} \min\sum_{i=1}^{N}\left\|\beta \cdot h(x_{i})-y_{i}\right\|\\ \min\left\|\beta \right\| \end{array}.$$

Therefore,

(9) $$\overset{⌢}{\beta }=H^{+}Y,$$

where, $H^{+}$ is the Moore–Penrose pseudo inverse of matrix *H*. Details can be found in Huang et al. [53].

To improve the generalization ability and reduce possible over-fitting problems, the training error is not supposed to be equal to zero. Therefore, the objective function can be rewritten as:

(10) $$\{\begin{array}{c} \min\sum_{i=1}^{N}\frac{1}{2}{\left\|\beta \right\|}^{2}+C\sum_{i=1}^{N}\xi_{i}\\ \mathrm{subject}\;\mathrm{to}\;h(x_{i})\beta =y_{i}^{T}-\xi_{i}^{T}\;i=1,2,\cdots Ν \end{array},$$

where, $\xi_{i}$ is the training error of the *i*th input vector, *C* is the regularization parameter. The optimal value of *β* can be obtained as:

(11) $$\overset{⌢}{\beta }=H^{T}(\frac{I}{C}+HH^{T}{)}^{-1}Y.$$

If h(x) is unknown, the Mercer’s conditions are introduced to the ELM model, a kernel function is defined as:

(12) $$K(x_{i},x_{j})=<h(x_{i}),h(x_{j})>.$$

Therefore, the output function can be rewritten as:

(13) $$f_{L}(x)=h(x)\cdot \beta =h(x)H^{T}(\frac{I}{C}+HH^{T}{)}^{-1}Y={\left[\begin{array}{c} K(x,x_{j})\\ \vdots \\ K(x,x_{N}) \end{array}\right]}^{T}{\left(\frac{I}{C}+\sum_{i,j=1}^{N}K(x_{i},x_{j})\right)}^{-1}Y.$$

The regularization parameter *C* is usually calculated by using n-fold cross-validation (CV) method [56]. From the Equation (13), it is shown that the output function is determined by the kernel function. There are several popular kernel functions, such as: polynomial functions, radial basis functions and so on. Different kernel functions are appropriate for different situations.

### 2.3. Riemannian Metric of Kernel-ELM

For classification, KELM supposes to find an optimal separating hyperplane, which passes through the origin of the KELM random feature space [55]. To modify the kernel function data-dependently, information geometry is employed to analyze the structure of kernel mapping geometrically. According to information geometry [59], h(x) is considered as an embedding of the input space *S* into the random feature space *F* as a curved submanifold. The mapped pattern of *x* is *z*: z=h(x). It can be expressed in differential form:

(14) $$dz=\nabla h(x)\cdot dx=\sum_{i}\frac{\partial }{\partial x_{i}}h(x)\cdot dx_{i},$$

here, the squared length of $dz=(dz_{\alpha })$ can be described as:

(15) $$\left|dz\right|=\sum_{\alpha }{\left(dz_{\alpha }\right)}^{2}=\sum_{i,j}g_{ij}(x)dx_{i}dx_{j},$$

where:

(16) $$g_{ij}(x)=(\frac{\partial }{\partial x_{i}}h(x))\cdot (\frac{\partial }{\partial x_{j}}h(x)).$$

$G(x)=(g_{ij}(x))$ is the Riemannian metric tensor in the input space. It shows that G(x) is a positive-definite matrix. According to the theorems introduced by Wu et al. [65], $g_{ij}(x)$ can be described as:

(17) $$g_{ij}(x)=\frac{\partial }{\partial x_{i}}\frac{\partial }{\partial {x^{\prime }}_{j}}K(x,x^{\prime })|{}_{x^{\prime }=x}={\left(\frac{1}{2}\frac{\partial^{2}K(x,x)}{\partial x_{i}\partial x_{j}}-\frac{\partial^{2}K(x,x^{\prime })}{\partial x_{i}\partial x_{j}}\right)}_{x^{\prime }=x}.$$

It is clear that the Riemannian metric *G*(*x*) can be directly determined by the kernel. Therefore, for Polynomial kernel, the induced Riemannian metric is:

(18) $$g_{ij}(x)=d\delta_{ij}+x_{i}x_{j}d(d-1).$$

For Gaussian radial basis function, the induced Riemannian metric is:

(19) $$g_{ij}(x)=2\gamma \cdot \delta_{ij},$$

where:

(20) $$\delta_{ij}=\{\begin{array}{cc} 1 & i=j\\ 0 & i\neq j \end{array}.$$

Therefore, the structure of kernel-ELM can be analyzed geometrically by using the Riemannian metric.

### 2.4. Information Geometrical Kernel-ELM

To modify KELM self-adaptively, we have to improve the generalization ability of KELM by using data rather than prior knowledge or expertise. From the perspective of the geometry, it can be found easily that the improvement of the spatial resolution around the optimal hyperplane will enhance the separability of patterns. In this study, a conformal transformation is utilized:

(21) $$\overset{⌢}{K}(x,x^{\prime })=D(x)D(x^{\prime })K(x,x^{\prime }),$$

where $\overset{⌢}{K}(x,x^{\prime })$ is the modified kernel function and D(x) is a positive scalar function. The new Riemannian metric ${\overset{⌢}{g}}_{ij}(x)$ can be rewritten as:

(22) $${\overset{⌢}{g}}_{ij}(x)=D{\left(x\right)}^{2}g_{ij}(x)+D_{i}(x)D_{j}(x)+2D_{i}(x)D(x)K(x,x),$$

where:

(23) $$\{\begin{array}{c} D_{i}(x)=\partial D(x)/\partial x_{i}\\ K_{i}(x,x)=\partial K(x,x^{\prime })/\partial x_{i}|{}_{x^{\prime }=x} \end{array}.$$

The selection of the factor D(x) should follow the rule that its value is greater when *x* is approaching to the boundary, and smaller when *x* is further away from the boundary. Therefore, the spatial resolution around the optimal hyperplane is enhanced.

However, the position of the hyperplane is unknown in practice. To solve this problem, we utilize Mahalanobis distance (MD) to estimate the approximate position of the hyperplane. The MD can be described as:

(24) $$MD_{i}^{\left(k\right)}={\left(x_{i}-u^{k}\right)}^{T}\sum_{}^{-1}\left(x_{i}-u^{k}\right),$$

where, $MD_{i}^{\left(k\right)}$ represents the distance between $x_{i}$ and the kth pattern, $u^{k}$ and $\sum_{}^{-1}$ represents the mean vector and covariance matrix of the kth pattern.

In view of the aforementioned analysis, the conformal mapping D(x) is given as:

(25) $$D(x_{i})=\frac{1}{M}\sum_{k=1}^{M}\exp(-\frac{1}{MD_{i}^{\left(k\right)}}),$$

where, *M* is the number of patterns. It is shown that the chosen D(x) is directly derived from the data only and follows the aforementioned rule. By using this approach, this study improves the generalization ability of KELM self-adaptively based on the training data with no requirement of prior knowledge or expertise.

**Figure 2:**
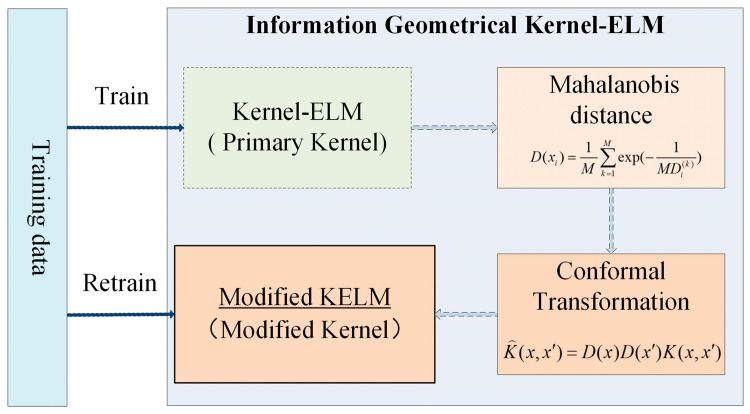
The scheme of IG-KELM.

In general, a novel algorithm called information geometrical Kernel-ELM (IG-KELM) is proposed, as shown in Figure 2:

1. Train the KELM with a primary kernel *K*;
2. Calculate Mahalanobis distances to obtain the conformal mapping D(x);
3. Transform the kernel K by Equation (21), and obtain the modified kernel $\overset{⌢}{K}$;
4. Retrain the KELM with the new kernel $\overset{⌢}{K}$.

### 2.5. Adaptive Diagnosis Based on VMD-SVD and IG-KELM

In this study, self-adaptive algorithms are used for both feature extraction and fault clustering; VMD-SVD is used for feature extraction and IG-KELM is used for fault clustering.

1. Feature extraction. VMD decomposes each signal into *n*-empirical modes (IMFs) self-adaptively, and the IMFs are constructed as a matrix. Then, SVD is employed to get a *n*-dimensional feature vector of singular values from the matrix.

2. Fault clustering. The proposed IG-KELM is employed for fault diagnosis under variable conditions.

The proposed method is self-adaptive and has strong robustness. Those advantages imply that this method is able to diagnose fault under unknown operation conditions without corresponding training data. The proposed method can be implemented self-adaptively under variable conditions, with little requirement of prior knowledge, expertise, parameter configuration or any other human intervention.

## 3. Simulation Experiment for IG-KELM

A comparison of simulation experiments between IG-KELM and the conventional KELM was made to verify the efficiency and self-adaptivity while using different kernel types and parameters.

### 3.1. Simulation Data

Suppose that there is a simulated two-dimensional dataset Z=(x,y) which is distributed evenly in the region [−2, 2] × [−2, 2]. The datasets are divided into two classes using a curve determined by $y=\sin(x)+\frac{\sin(3x)}{3}-2\sin(\frac{x}{2})$. Then, the IG-KELM or KELM produces a new boundary to cluster the dataset. The accuracy of the classification can be employed to verify the efficiency and self-adaptivity of IG-KELM or KELM while using different kernel functions and parameters.

**Figure 3:**
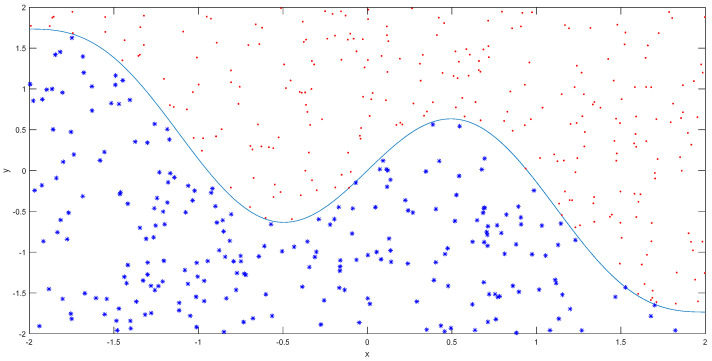
500 training samples of simulation experiment.

In this simulation, 500 training samples were randomly and uniformly generated, as shown in Figure 3. Another 2000 test samples were generated for verification. Two types of kernel functions were involved, Gaussian RBF and Polynomial kernels in this study. In IG-KELM.

### 3.2. Simulation Results

#### 3.1.1. By Using the Gaussian RBF Kernel

**Figure 4:**
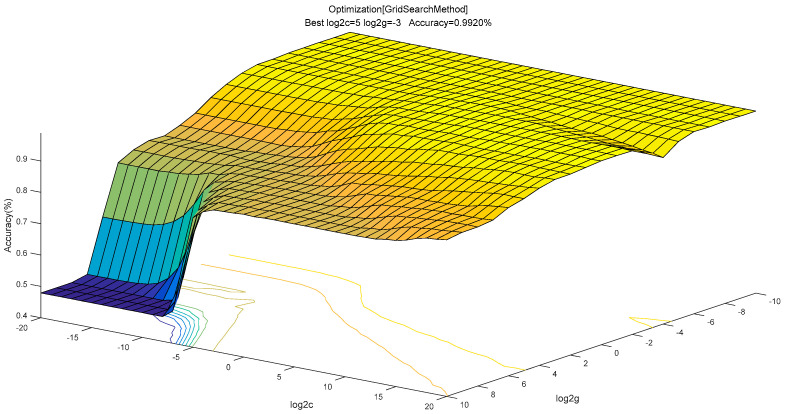
Optimization of parameters by using 5-fold cross-validation (CV) method.

As aforementioned, 500 training data were used to train the KELM with a Gaussian RBF kernel. According to Huang et al. [56], the regularization parameter *C* can be selected from [2^−20^, 2^−19^, … , 2^19^, 2^20^], and kernel parameter γ can be selected from [2^−10^, 2^−9^, …, 2^9^, 2^10^]. A five-fold cross-validation (CV) method is employed to optimize the parameters, as shown in Figure 4. As a result, the optimal C and γ were 2^5^ and 2^−3^ respectively.

Then, the classification results of KELM and IG-KELM were calculated, as shown in Table 1. The results show that the test accuracy of KELM is 99.20%, while the test accuracy of IG-KELM is 99.65%. Therefore, the trained IG-KELM performed better than KELM.

**Figure 5:**
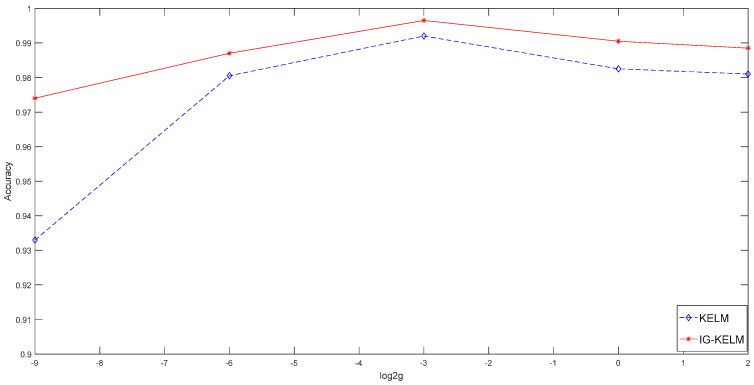
Comparison of test accuracy rates of KELM (blue curve) and IG-KELM (red curve) with the RBF kernel.

The classification results were also calculated when $\gamma =2^{-9},2^{-6},2^{0},2^{2}$ to verify whether the IG-KELM can perform acceptably even if the kernel parameter is set badly, as shown in Table 1 and Figure 5. In general, when the value of γ is away from the optimal value ($\gamma =2^{-3}$), the accuracy of KELM dropped obviously. However, the IG-KELM always performed well. Especially, if *γ* was set to 2^2^, the test accuracy rate of KELM dropped to about 93%, while the IG-KELM still performed at a high level (97.40%). The results show that the IG-KELM is insensitive to inappropriate parameter configuration, compared with KELM.

#### 3.1.2. By Using the Polynomial Kernel

**Figure 6:**
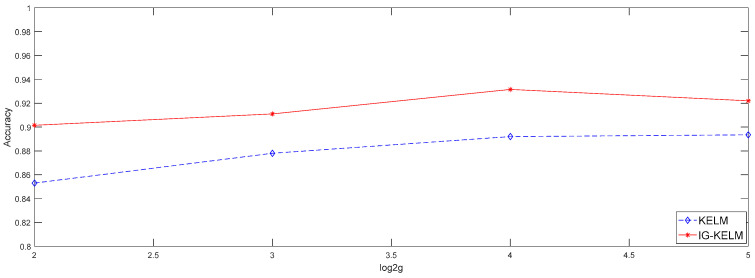
Comparison of test accuracy rates of KELM (blue curve) and IG-KELM (red curve) with the Polynomial kernel.

The simulation results of KELM and IG-KELM with a Polynomial kernel were obtained when d=2,3,4,5, as shown in Figure 6 and Table 2.

Since the polynomial kernel is obviously inappropriate for the dataset, all test accuracy rates of KELM are below 90%. However, the IG-KELM improves the performance rapidly even with a bad kernel type. When d was set to 4, the test accuracy rate of IG-KELM reached to 93.15%. It is obvious that the IG-KELM has strong robustness to the inappropriate kernel types, compared with KELM.

## 4. Application Cases of the Proposed Diagnosis Method

### 4.1. Application on Bearing Fault Diagnosis

Rolling bearing is one of the most wildly used component in rotating machineries. Therefore, the vibration data of bearings were used to verify the propose method.

#### 4.1.1. Experimental Setup of Bearing

**Figure 7:**
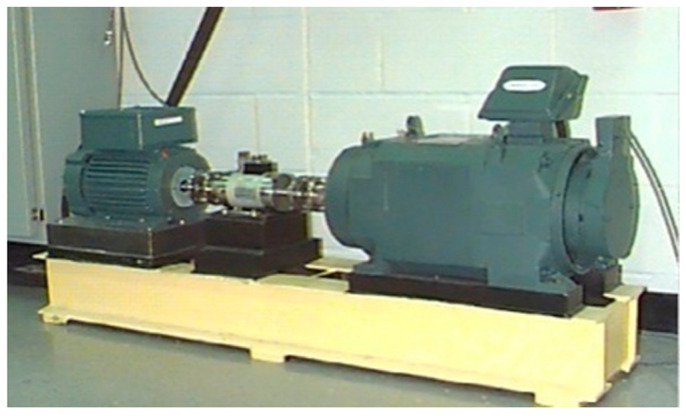
Experimental test rig in bearing data center.

The data from the bearing data center of Case Western Reserve University were used in this study. The test rig contains a 2 HP motor, a torque converter/encoder, a dynamometer and control electronics, and is shown in Figure 7 (More details about the test rig can be found in http://www.eecs.case.edu/laboratory/bearing/welcome_overview.htm.). The 6205-2RS JEM SKF deep-groove ball bearings were tested under four operation conditions (condition A, B, C, D), corresponding to different motor speeds and loads, as shown in Table 3. The vibration signals, including normal, inner race fault, outer race fault and rolling element fault signals were collected with a sampling rate of 12 kHz under every condition. There are four groups of data (group A, B, C, D), corresponding to four operation conditions. Each of four groups contains 50 training samples and 100 test samples, as shown in Table 3. To verify the proposed diagnosis method under unknown variable conditions, we trained four models corresponding to four conditions respectively, and tested each of them under every condition without any prior knowledge or human intervention.

#### 4.1.2. Feature Extraction Based on VMD-SVD

**Figure 8:**
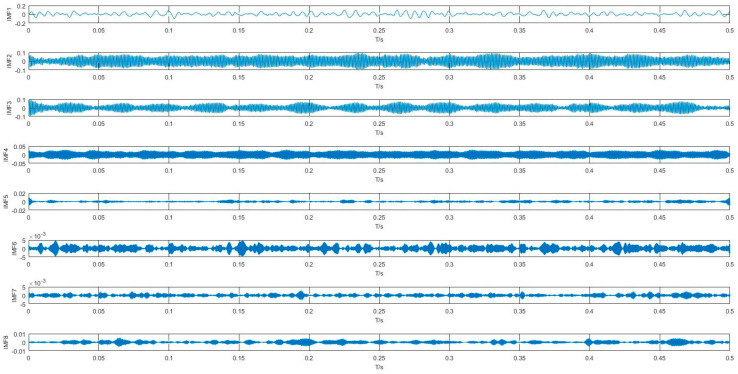
IMFs obtained by VMD from the normal signal.

**Figure 9:**
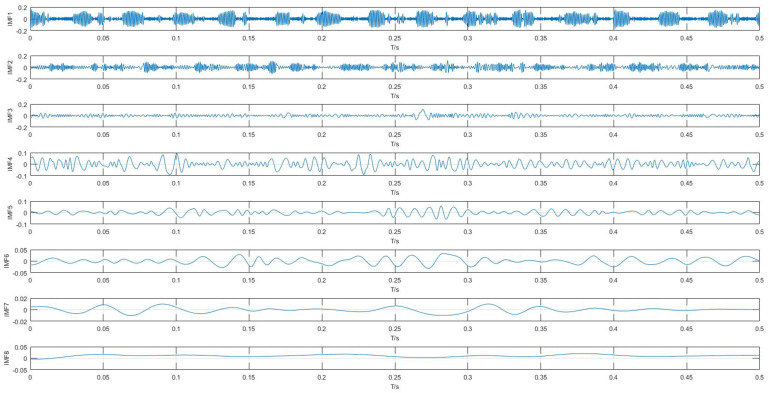
IMFs obtained by EMD from the normal signal.

Each data sample was decomposed into n IMFs by VMD. In this study, n was set to 8 by default. Take a normal signal for example, the processing result is shown in Figure 8. For comparison, the results by EMD was also obtained, as shown in Figure 9. Compared with results of VMD and EMD, it is shown that VMD can separate signals more effectively and eliminate the modal aliasing problem.

**Figure 10:**
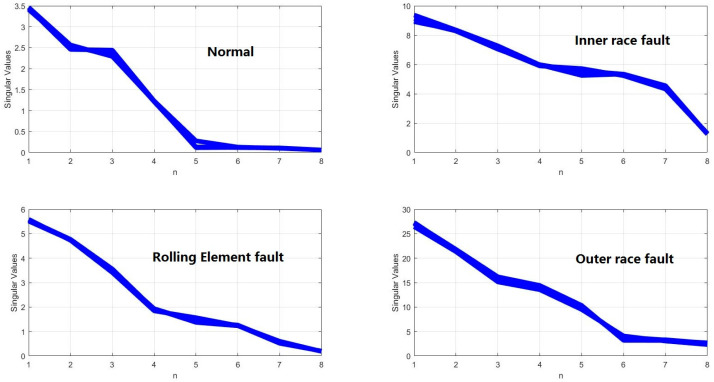
Singular values obtained by VMD-SVD.

**Figure 11:**
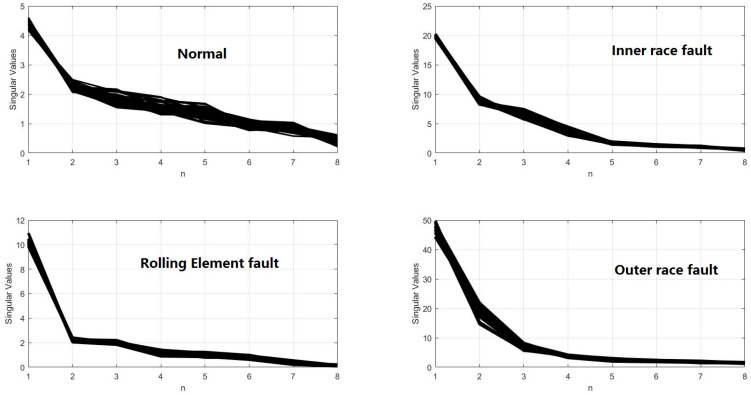
Singular values obtained by EMD-SVD.

The IMFs were used to construct the matrix. Then, the singular values can be acquired by using SVD. Take Condition A for example, the results are shown in Figure 10. Compared with results of EMD-SVD (as shown in Figure 11), for each certain fault mode, the results obtained by VMD-SVD are more coincident and stable, respectively. Different fault modes can be easily distinguishable by using VMD-SVD. On the contrary, the results of EMD-SVD show more variability. That is obviously a disadvantage to fault diagnosis.

#### 4.1.3. Fault Clustering for Bearing

In this study, IG-KELM with an RBF kernel was employed for fault clustering based on features extracted by VMD-SVD. Four trained models, corresponding to four conditions were obtained and tested under every operation condition, respectively, as shown in Table 4. The conventional KELM was employed for comparison.

In this study, all training errors of KELM and IG-KELM models were calculated to be zero; if the trained model and test samples come from the same condition, all test accuracy rates are 100% (which means the test errors are zero). That is mainly due to the efficiency of the hybrid feature extraction method (VMD-SVD). However, if the trained model and test samples come from different conditions, the test accuracy rates of KELM decrease rapidly. When the trained KELM from Condition B was employed under Condition D, the test accuracy rate was only 89.25%, which is unacceptable in applications.

On the contrary, IG-KELM performed much better under unknown variable conditions and the test accuracy rates were no less than 96%. Compared with the strong sensitivity of KELM to the operation condition, the IG-KELM is able to adapt itself in a data-dependent way and improves the performance under unknown variable conditions rapidly. The IG-KELM can be implemented even without any prior knowledge about the current operation condition.

Therefore, the novel method using IG-KELM based on VMD-SVD can be utilized for bearing fault diagnosis under unknown variable conditions with little prior knowledge or human intervention.

### 4.2. Application on Hydraulic Pump Fault Diagnosis

**Figure 12:**
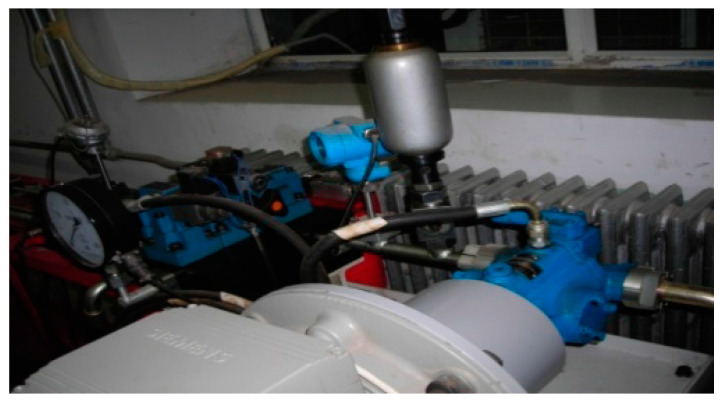
Experimental test rig of SCY hydraulic plunger pump.

The proposed method was also applied on fault diagnosis of a hydraulic pump. The vibration signals were gathered from a test rig of SCY hydraulic plunger pump with a sampling rate of 1000 Hz, as shown in Figure 12. The nominal pressure is 31.5 Mpa, and the nominal displacement is 1.25–400 mL/r. The pump was running at a fluctuant motor speed of 5280 ± 200 rpm while gathering signals. Vibration signals were collected using a four-channel DAT recorder. Considering that the most crucial fault modes of the plunger pump are slipper loosing and valve plate wear, the dataset consists of three types of states, corresponding to no trouble (Normal), slipper loosing (Fault 1) and valve plate wear (Fault 2), as shown in Table 5. 

The proposed method was applied on the dataset and KELM was also used for comparison, as shown in Table 6, from which we can see that KELM has a high false alarm rate (25/30). That is because some normal data under unknown fluctuant conditions were identified as abnormal data by the KELM model. On the contrary, the IG-KELM has strong robustness to fluctuant conditions and can reduce the false alarms and improve the classification accuracy. The results show that the application of the proposed method on hydraulic pump fault diagnosis is feasible and efficient.

## 5. Conclusions

This paper focuses on adaptive diagnosis for rotating machineries, which can diagnose faults automatically under unknown variable operation conditions with little prior knowledge or human intervention. For this end, this paper proposes a method using IG-KELM based on VMD-SVD for fault diagnosis. Firstly, the VMD–SVD method is employed to extract features from the vibration signals self-adaptively. Secondly, IG-KELM, which employs information geometry to modify KELM data-dependently is used for fault clustering. The IG-KELM can be modified self-adaptively, and be insensitive to the manual configuration of kernel. The simulation results show that IG-KELM can increase the accuracy rate by up to 4.85%. Therefore, IG-KELM can perform efficiently even if the kernel types or parameters are set badly. Finally, the proposed method was applied on fault diagnosis of bearing and hydraulic pump under variable conditions. Compared with the conventional method, the proposed method increased the accuracy rates by up to 7.25% and 7.78%, respectively. The results show that the proposed method has strong self-adaptivity and high accuracy during feature extraction and fault clustering.

Considering that this proposed method can be applied automatically and require little prior knowledge or human intervention, it can be available on digital signal processors (DSPs), field programmable gate arrays (FPGAs) or even smartphones.

However, to some extent, the operation conditions involved in this study only fluctuated in a narrow region. Larger differences of operation conditions may create new problems. Therefore, additional experiments under more different conditions should be done to validate and improve the method. Meanwhile, more attentions should be paid to the development of more and better adaptive diagnosis methods.

## Figures and Tables

**Table 1:** Simulation experiments of KELM and IG-KELM with an RBF kernel.

	KELM			IG-KELM		
	Training Error Rate	Test Error Rate	Test Accuracy	Training Error Rate	Test Error Rate	Test Accuracy
$\gamma =2^{2}$	3.60%	6.70%	93.30%	1.60%	2.60%	97.40%
γ=0	0.80%	1.95%	98.05%	0.60%	1.30%	98.70%
$\gamma =2^{-3}$	0.20%	0.80%	99.20%	0.20%	0.35%	99.65%
$\gamma =2^{-6}$	0.60%	1.75%	98.25%	0.20%	0.95%	99.05%
$\gamma =2^{-9}$	1.00%	1.90%	98.10%	0.40%	1.15%	98.85%

**Table 2:** Simulation experiments of KELM and IG-KELM with a Polynomial kernel.

	KELM			IG-KELM		
	Training Error Rate	Test Error Rate	Test Accuracy	Training Error Rate	Test Error Rate	Test Accuracy
d=2	9.40%	14.70%	85.30%	4.20%	9.85%	90.15%
d=3	8.00%	12.20%	87.80%	3.40%	8.90%	91.10%
d=4	7.80%	10.80%	89.20%	2.00%	6.85%	93.15%
d=5	6.40%	10.65%	89.35%	2.40%	7.80%	92.20%

**Table 3:** Experimental datasets of bearing fault diagnosis.

Operation Condition	Motor Speed (rpm)	Motor Load (HP)	Normal		Inner Race Fault		Outer Race Fault		Rolling Element Fault	
			Training	Test	Training	Test	Training	Test	Training	Test
A	1797	0	20	40	10	20	10	20	10	20
B	1772	1	20	40	10	20	10	20	10	20
C	1750	2	20	40	10	20	10	20	10	20
D	1730	3	20	40	10	20	10	20	10	20
Total			80	160	40	80	40	80	40	80

**Table 4:** Results of bearing fault diagnosis.

Operation Condition	Model	Operation Condition			
		A	B	C	D
		Accuracy	Accuracy	Accuracy	Accuracy
A	KELM	100%	99.50%	99.25%	99.25%
	IG-KELM	100%	100%	100%	100%
B	KELM	94.75%	100%	99.75%	89.25%
	IG-KELM	96.50%	100%	100%	96.50%
C	KELM	93.00%	98.50%	100%	91.50%
	IG-KELM	97.75%	99.50%	100%	98.25%
D	KELM	96.50%	97.75%	92.75%	100%
	IG-KELM	97.75%	100%	98.50%	100%

**Table 5:** Experimental datasets of hydraulic pump fault diagnosis.

Type	SCY Plunger Pump			Total
	Normal	Slipper Loosing	Valve Plate Wear	
No. of training samples	30	30	30	90
No. of test samples	30	30	30	90

**Table 6:** Results of hydraulic pump fault diagnosis.

Model	KELM			IG-KELM		
	Training Error	Test Error	Test Accuracy	Training Error	Test Error	Test Accuracy
Normal	3/30	5/30	25/30	0/30	0/30	30/30
Slipper loosing	0/30	1/30	29/30	0/30	0/30	30/30
Valve plate wear	1/30	3/30	27/30	0/30	2/30	28/30
Total	4/90	9/90	81/90	0/90	2/90	88/90
Percentage	4.44%	10.00%	90.00%	100%	2.22%	97.78%
